# Synthesis, Interfaces, and Nanostructures: A Section of *Nanomaterials* (ISSN 2079-4991)

**DOI:** 10.3390/nano11112850

**Published:** 2021-10-26

**Authors:** Paolo Scrimin

**Affiliations:** Department of Chemical Science, University of Padova, via Marzolo, 1, 35131 Padova, Italy; paolo.scrimin@unipd.it; Tel.: +39-049-827-5276

“Synthesis, Interfaces, and Nanostructures” is one of the pillar sections of *Nanomaterials* and has contributed to the significant increase in the journal’s recognition by the scientific community, boosting its Impact Factor to 5.076.

When this Section was introduced two years ago (in 2019), we wrote that it was designed for manuscripts “dealing with the chemistry at interfaces reporting on phenomena such as, for example: adsorption, reactions, films, forces, measurement techniques, charge transfer, electrochemistry, electrocatalysis, energy production and storage but also discussing systems presenting interfacial regions. These include nanoparticles, colloids, emulsions, surfactants, proteins, polymers as non-exhaustive examples.” While the basic scope of the Section is unchanged, the science has evolved, focusing more on applications and catalysis. For this reason, we encourage colleagues dealing with these topics to consider *Nanomaterials* for the publication of their results. The importance of renewable energies calls for intensive research, aiming to provide solutions to the demand for replacing fossil energy sources. Nanostructures constitute valuable options in this regard, with very important results having been achieved [1]. Catalysis significantly contributes to more efficient transformations with enhanced selectivity. Natural enzymes have stimulated the development of “nanozymes” [2,3], a possible answer to the quest for powerful, man-made catalysts. One of the “hot” topics in green energy conversion is water-splitting, in which nanoparticles could play an important role [4,5].

All manuscripts that are submitted for publication in this section will be reviewed by our expert Section Editors and undergo a rigorous peer-review process. We aim for quality over quantity. Our goal is to offer our readers free-to-read, high-quality papers reporting cutting-edge research in nanomaterials within the broad field of this section. We look forward to receiving your next important manuscript!
